# Planar Indium Tin Oxide Heater for Improved Thermal Distribution for Metal Oxide Micromachined Gas Sensors

**DOI:** 10.3390/s16101612

**Published:** 2016-09-29

**Authors:** M. Cihan Çakır, Deniz Çalışkan, Bayram Bütün, Ekmel Özbay

**Affiliations:** 1 Nanotechnology Research Center, Bilkent University, Ankara 06800, Turkey; dcaliskan@fen.bilkent.edu.tr (D.Ç.); bbtn@bilkent.edu.tr (B.B.); ozbay@bilkent.edu.tr (E.Ö.); 2 Department of Nanotechnology and Nanomedicine, Hacettepe University, Ankara 06800, Turkey; 3 Department of Electrical and Electronics Engineering, Department of Physics, Bilkent University, Ankara 06800, Turkey

**Keywords:** Indium tin oxide, Metal oxide gas sensor, Micro hot-plate, SnO_2_, Heat distribution

## Abstract

Metal oxide gas sensors with integrated micro-hotplate structures are widely used in the industry and they are still being investigated and developed. Metal oxide gas sensors have the advantage of being sensitive to a wide range of organic and inorganic volatile compounds, although they lack selectivity. To introduce selectivity, the operating temperature of a single sensor is swept, and the measurements are fed to a discriminating algorithm. The efficiency of those data processing methods strongly depends on temperature uniformity across the active area of the sensor. To achieve this, hot plate structures with complex resistor geometries have been designed and additional heat-spreading structures have been introduced. In this work we designed and fabricated a metal oxide gas sensor integrated with a simple square planar indium tin oxide (ITO) heating element, by using conventional micromachining and thin-film deposition techniques. Power consumption–dependent surface temperature measurements were performed. A 420 °C working temperature was achieved at 120 mW power consumption. Temperature distribution uniformity was measured and a 17 °C difference between the hottest and the coldest points of the sensor at an operating temperature of 290 °C was achieved. Transient heat-up and cool-down cycle durations are measured as 40 ms and 20 ms, respectively.

## 1. Introduction

Metal oxides are widely used in gas sensing applications, such as chemo-resistive [1] and optoelectronic sensors [2,3]. Metal oxide–resistive gas sensors are being investigated, developed, and used due to their simplicity, production flexibility, low cost and sensitivity to a wide variety of volatiles [1]. Due to increasing interest in those sensors, micro-hotplate structures are being developed with enhanced device performance, decreased power consumption, and low cost [1,4]. Research activities aimed at enhancing the sensitivity and selectivity as well as lowering the response time and power consumption are still under active research. Those research studies present innovative integrated micro-hotplate technologies based on Micro Electro Mechanical Systems (MEMS) to achieve optimized thermal properties such as low power consumption and good temperature uniformity across the active layer [4].

With the development of MEMS technologies in the 1990s, new generations of gas sensors with integrated micro-hotplates were realized. Micromachining processes allow for the precise modification of the thermal characteristics of those devices. The optimization of device geometry, membrane, and resistor materials affects the heat transfer mechanisms, resulting in the enhancement of the thermal dispersion and Joule heating characteristics of the devices. Besides these enhancements, the sensor’s heat capacity reduction, which is achieved by thermal mass reduction with the help of micromachining methods, also reduces the power consumption and improves the response time of the sensors [5,6]. By improving the response time, selectivity could be enhanced by rapid temperature cycle measurements to produce additional information by getting the response signature as a variable of temperature pulses [7,8,9].

Metal oxide gas sensors are based on the surface reactions between the target gas species and the sensing metal oxide film. As a result of the surface reactions, gas molecules interact with the film surface and dope it, the surface band bending alters and then the metal oxide layer resistivity changes.

Metal oxide sensors give a response to the wide variety of organic and inorganic gases. However, this wide range has a disadvantage, which is nonselectivity. This disadvantage is eliminated once a temperature-dependent measurement is used. When gas species are introduced to the film surface, the total amount of surface reactions, i.e., the adsorbed and desorbed amount of gas molecules, is correlated with the resistance change of the thin film, and it is temperature-dependent and specific to the gas compound type. The base conductance of the sensing film can be defined as the conductance in the absence of the target gas molecules at a given temperature, and it is also strongly dependent on the temperature of the sensing film [6]. Different metal oxides react with the same analytes with different efficiencies at different temperatures [6,10,11]. In other words, the absolute resistivity change caused by the same concentration of the target gas can reach its peak value at different temperatures for different metal oxide films. This information can be used to obtain more selective data extraction from metal oxide gas sensors.

For a given temperature-dependent measurement, the nonuniformity of the surface temperature distribution means that the total sensor signal will be an integration of signals obtained from metal oxide sites at different temperatures. Larger nonuniformity in temperature means that the data will be blurred and the peaks in the resistance change with temperature changes will be broadened. To get a characteristic temperature-dependent sensor signal at a high resolution, the temperature distribution on the active sensing film region has to be as uniform as possible [12,13,14].

To reach good temperature uniformity, power consumption, and working temperature goal by using conventional metal and metal alloy resistor materials, the hotplates with heat-spreading plates, micro-bridges, and resistors with complex geometries based on meanders were designed and fabricated [4,5,6,14,15].

## 2. Design and Fabrication

In this work, a planar resistor as a heating element is introduced which can provide high temperature uniformity across the active region of the thin-film metal oxide sensors. A thin sputtered indium tin oxide (ITO) film is used as the resistor material, with simple planar resistor geometry. Silicon nitride (Si_3_N_4_) is used for both the membrane material and also as the passivation and insulation layers. Conventional bulk silicon micromachining methods such as potassium hydroxide (KOH) etching are used to obtain the micro-hotplate structures. In order to make a realistic thermal characterization, not only a bare micro-hotplate structure is measured; we also added thermal mass on the hotplate structure by depositing the contact metals for the sensing metal oxide thin film and the metal connection pads by e-beam evaporation and the thin tin oxide (SnO_2_) metal oxide sensing layer by sputtering.

**Figure 1:**
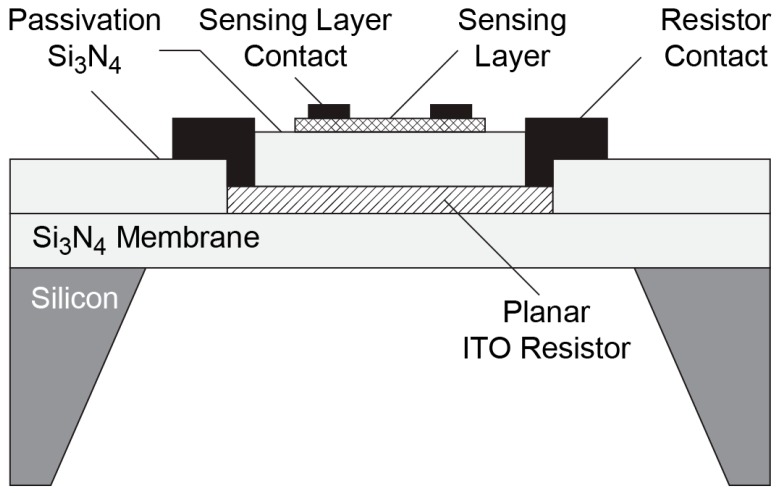
Cross-section of the gas sensor.

The structure of the metal oxide gas sensor is shown in Figure 1, which demonstrates the cross-section of the gas sensor. As shown in Figure 1, the basic concept of the hotplate is the planar-structured, continuous ITO resistor that lies underneath the active metal oxide layer, isolated with a thin Si_3_N_4_ layer.

Sensors were fabricated on p-type, double-side, polished, 300-µm-thick (100) silicon (Si) substrates. As the first step of the fabrication, 1.8 µm thermal SiO_2_ is grown on both sides of a wafer in a tube-furnace (MTI, Richmond, CA, USA) using wet oxidation. This layer serves as an extra masking layer during the bulk Si etching process and also as a mechanical protection layer for the fabrication steps on the backside of the silicon substrate, avoiding scratches. A 1-µm-thick Si_3_N_4_ layer is deposited on the thermal SiO_2_ with plasma-enhanced chemical vapor deposition (PECVD) (Samco, Kyoto, Japan) to form an etch stop layer for the wet anisotropic Si etching process. Si_3_N_4_ is preferred due to its very low etch rate in KOH solution.

**Figure 2:**
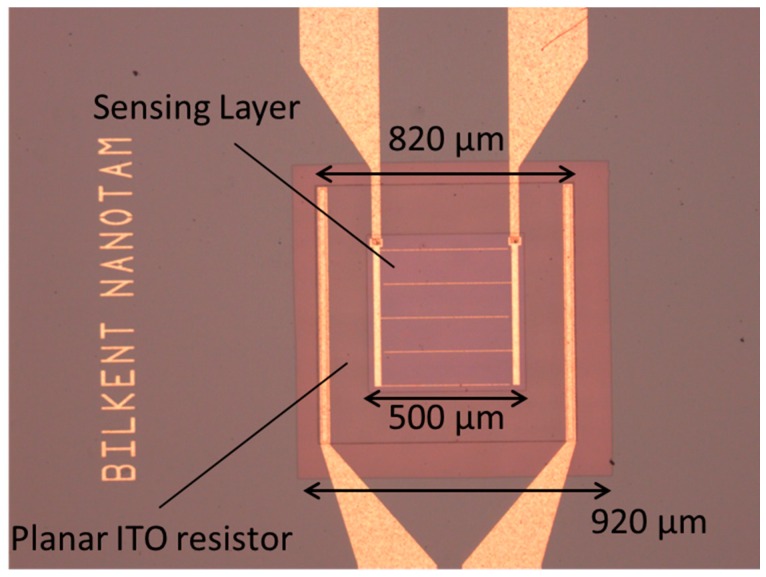
Fabricated sensor’s optical microscope photograph.

The thermal oxide on the front side of the wafer is removed by reactive ion etching in an inductively coupled plasma reactor (ICP-RIE) (Samco, Kyoto, Japan) using fluorine chemistry. The etching parameters were 75 W ICP, 100 W RF bias power, 0.25 Pa chamber pressure, and 30 sccm CHF_3_ process gas flow. Then, 1-µm-thick Si_3_N_4_ is grown with PECVD as a membrane of the sensor. PECVD Si_3_N_4_ was preferred due to its low thermal conductivity [3] in order to reduce the heat losses due to thermal conduction. Si_3_N_4_ deposition parameters were 130 W RF power, 275 °C temperature, 75 Pa chamber pressure and precursor gas flow was SiH_4_ (160 sccm)/NH_3_ (6 sccm)/N_2_ (500 sccm). Resistor patterns are defined by reversal photolithography. The ITO resistor is deposited by RF magnetron sputtering system (Leybold Oerlikon, Köln, Germany) using an indium oxide/tin oxide (90/10 by wt. %) target with a 99.99% purity. Deposition is performed at 75 W RF power, 2.2 × 10^−3^ mbar pressure with a rate of 1.4 Ȧ/s in Ar atmosphere resulting in an ITO layer thickness of 140 nm. After deposition, lift off is done. The 1-µm-thick Si_3_N_4_ is grown with PECVD as a passivation layer. The bulk silicon etch mask is defined by photolithography and patterned into Si_3_N_4_ by ICP-RIE. The openings on the passivation are etched with ICP-RIE by using CHF_3_ plasma. A 200-nm-thick SnO_2_ is deposited as an active layer with RF magnetron sputtering system (Nanovak, Ankara, Turkey) by using the SnO_2_ sputtering target with 99.99% purity. Prior to the active material deposition, Cr/Au/Pt contact metals are deposited by e-beam evaporation as resistor contacts. Cr/Au thick metal contact pads for the sensing layer are deposited with an e-beam evaporator system (Leybold Oerlikon, Köln, Germany) as well. Bulk silicon etch was performed in 33% KOH solution at 90 °C for the formation of the membrane by using a special wafer holder with backside protection capability. The microscope photograph of the final sensor device is shown in Figure 2. The total heated area, which is also the ITO resistor area, was 820 × 820 µm^2^ while the overall membrane area was 920 × 920 µm^2^. The SnO_2_ active sensing area was 500 × 500 µm^2^ and positioned geometrically at the center of both the membrane and ITO micro-heater.

## 3. Results and Discussion

**Figure 3:**
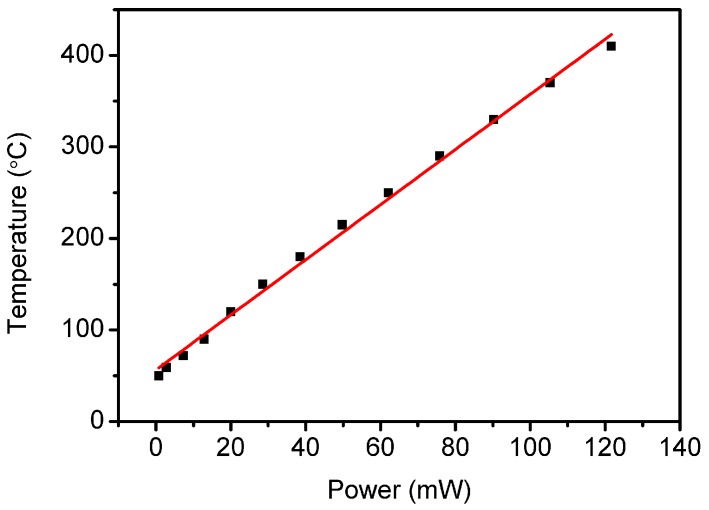
Power consumption versus measured maximum metal oxide film temperature.

The resistance of the square-shaped ITO heater resistor was measured as 35 Ohms by the four-point resistance measurement technique. By using a thermal microscope, the relationship between the input power and active area temperature was measured, as shown in Figure 3. The calibration temperature used for the measurements was 50 °C. The graph implies that the heating efficiency of the structure is weakly dependent on the temperature. At higher heating powers, the heating efficiency decreases slightly. The slope of the linear fitting line gives the heating efficiency of the sensor as 3 °C/mW.

Temperature mapping is performed with QFI InfraScope II (Quantum Focus Istruments, Vista, CA, USA), with a liquid nitrogen–cooled InSb detector, using radiance and uniformity calibration targets. Measurement calibration is done by keeping the sample on the microscope stage with a high thermal conductance liquid between the surfaces. The stage temperature is fixed at 50 °C, no bias is applied to the micro-heater and the reference radiance is taken. This way, different emissivity values of the metal electrodes, ITO heater and dielectric coated areas are calibrated using measured radiance data as references. Then bias is applied to the micro-sensor, and then temperature distribution uniformity is measured and corrected according to the reference map.

**Figure 4:**
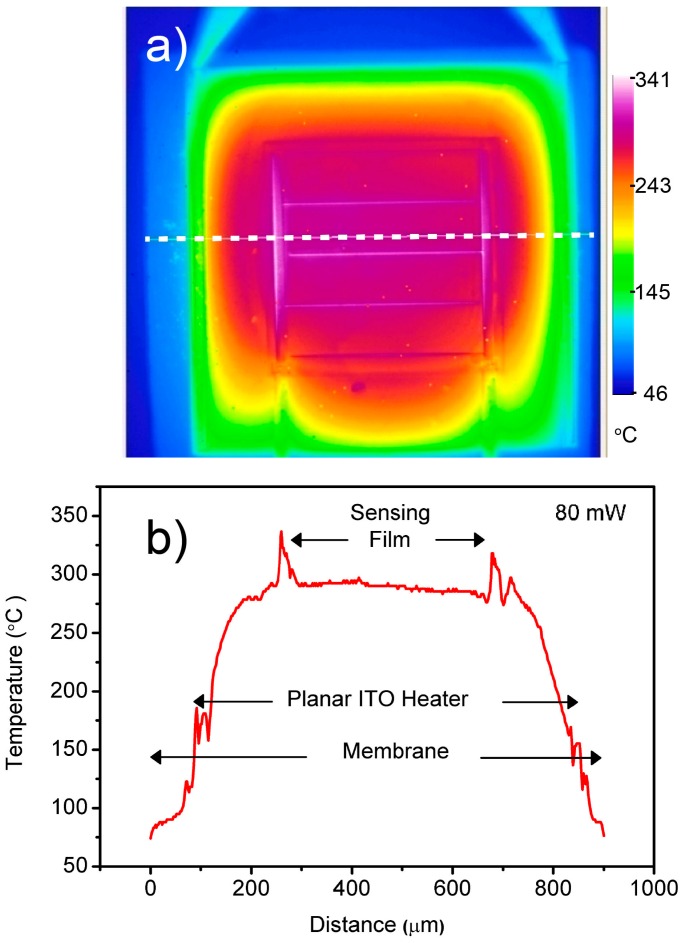
Temperature distribution image taken by a thermal microscope (**a**) and temperature distribution line scan through center of the device at 80 mW (**b**).

Figure 4 shows the thermal microscope view of the operating device at a 80 mW heating power. As can be seen from the line scan through the center of the heated zone, the temperature difference between the planar resistor contacts is about 30% of the maximum temperature at the center of the heater. However, the temperature change in the SnO_2_ active layer area was measured to be lower than 7% with a maximum 17 °C temperature difference between the hottest and coldest points. Sudden temperature jumps on the sensor electrodes and heater electrodes were probably linked to the temperature dependence of the metal emissivities, whereas the thermal microscope uses a single emissivity value for each pixel at the reference radiance measurement temperature.

**Figure 5:**
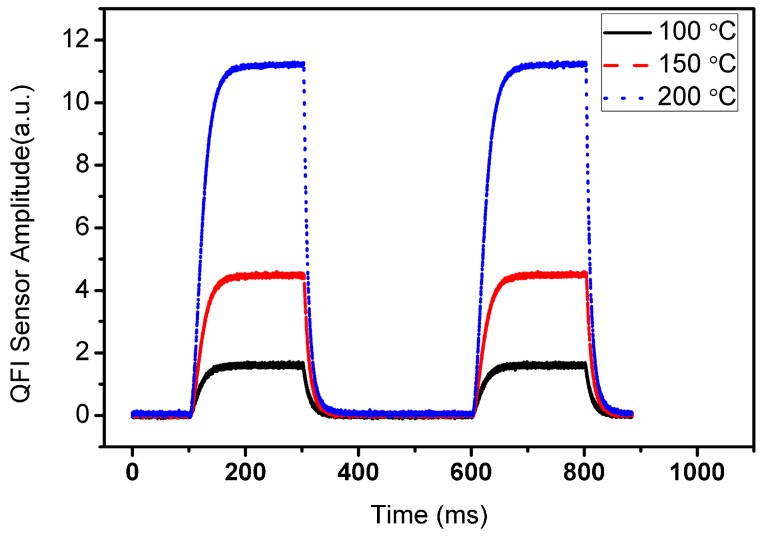
Heat-up and cool-down times as measured at 100 °C, 150 °C, and 200 °C average active area temperature.

Figure 5 shows the results of transient measurements taken with the thermal microscope. Measurements were performed at 2 Hz with a pulse width of 200 ms.

As is commonly used, the rise time can be defined as the time for the sensor to heat up from 10% to 90% of the maximum stabilized temperature. Similarly, the fall time is defined as the time for the sensor to cold down from 90% to 10% of its maximum temperature. Transient measurements were made for three different working temperatures. Heat-up and cool-down times of 40 ms and 20 ms were obtained, respectively. These values can be improved by reducing the size of the heater areas inside the thermally insulating Si_3_N_4_ membranes to reduce heat leakages. Also, by using a low-pressure PECVD (LPECVD) membrane and passivation layers with low intrinsic stress, the film thicknesses can be further reduced. In this way the thermal mass can be reduced and the rise-fall time values can be improved.

## 4. Conclusions

This work describes the design and fabrication of a novel micro-hotplate structure for thin-film metal oxide gas sensors, using silicon micromachining methods. ITO is used as the resistor material with a square planar geometry. The structure and fabrication techniques are simple but they meet the required performance specifications of low power consumption and linearity of heating efficiency for such devices. Further, the demonstrated heating element achieves high temperature uniformity with a 17 °C gradient at a 300 °C working temperature on the active film area with 80 mW power consumption. The rise and fall times are measured to be 40 ms and 20 ms, respectively. The hotplate structure with the square planar ITO geometry that we introduced solves the temperature nonuniformity problem. Cold spots at elevated temperatures, which arise from the non-continuous nature of conventional resistor meanders [14], are avoided. Key advantages of the planar ITO resistors are their continuity underneath the active metal oxide layers and the lacking necessity of extra microstructures, such as heat-spreading plates. The micro-hotplate with a planar ITO resistor can serve as the platform for integrated thin-film gas sensors. It brings simplicity to sensor design and fabrication. Both the membrane structure and the resistor are transparent; it could be a promising technology for sensor applications based on both of the electrical and optical measurements.
